# English Groom and Yankee

**Published:** 1874-01

**Authors:** 


WHAT AN ENGLISH GROOM SAID TO A YANKEE HOSTLER.—When a horse comes in all wet with prespiration, you let him stand in the stable and dry with all the dirt on. In England, we take the horse as he comes in from a drive and sprinkle blood-warm water all over him, from his head to his feet. Then we scrape him down and blanket him, rubbing his legs and face dry. Thus, in an hour, he is clean and dry, and ready to take a good feed, while with your way he will stand and swelter for hours, and finally dry sticky and dirty. Our horses never founder and never take cold. We never use a curry-comb. You scratch your horses too hard. The care necessary is to have the water not very cold, then bathe them instantly, while you are rubbing their legs.

